# Evaluation of Anorectal Function in Perianal Crohn’s Disease: A Pilot Study

**DOI:** 10.3390/jcm10245909

**Published:** 2021-12-16

**Authors:** Andreia Albuquerque, John Casey, Grace Fairlamb, Lesley A. Houghton, Christian Selinger

**Affiliations:** 1 Gastroenterology Department, St. James’s University Hospital, Leeds LS9 7TF, UK; christian.selinger@nhs.net; 2 GI Physiology Department, St. James’s University Hospital, Leeds LS9 7TF, UK; john.casey3@nhs.net (J.C.); grace.fairlamb@nhs.net (G.F.); 3 Leeds Institute of Medical Research, St. James’s University Hospital, Leeds LS9 7TF, UK; L.A.Houghton@leeds.ac.uk

**Keywords:** perianal Crohn’s disease, anorectal function, high-resolution anorectal manometry, balloon expulsion test, 3D-endoanal ultrasound

## Abstract

Background: Perianal Crohn’s disease is a disabling condition, with little known about anorectal function in healed/inactive perianal Crohn’s disease; Aim: To evaluate anorectal function in a cohort of patients with treated/healed perianal Crohn’s disease; Methods: Prospective cohort study, including high-resolution anorectal manometry, balloon expulsion test, and 3D-endoanal ultrasound in all patients; Results: Of the 16 patients studied (mean age ± SD, 42 ± 13 years), 12 (75%) were men. A laceration of the internal anal sphincter and/or anal scarring was seen in nine (56%) patients; there was no laceration of the external anal sphincter. Five (56%) of these nine patients had never experienced faecal incontinence. All had normal anal resting and squeeze pressures. Manometry suggested dyssynergia in 11 (69%) patients, with only one (6%) fulfilling the criteria for obstructed defecation. Hyposensitivity for at least one sensory parameter was seen in 11 (69%) patients and hypersensitivity in five (31%) patients; Conclusions: This study detected sphincter abnormalities in more than half of patients, many of whom were asymptomatic. Alterations in rectal sensation were frequently seen, more commonly with rectal hyposensitivity. Trial registration: ClinicalTrials.gov (NCT03819257).

## 1. Introduction

Perianal Crohn’s disease (CD) is a disabling disease associated with increased morbidity and impaired quality of life. It can be associated with pain, discharge, faecal incontinence, and sexual and psychological impairment [1]. A higher prevalence is seen with increasing disease duration and is related to disease location [2]. Studies have shown that the prevalence of perianal CD varies between 12% in small bowel disease, up to 92% in colonic disease with rectal involvement [2,3].

Anorectal manometry is the best technique for evaluation of anal sphincter pressures. It also allows for an evaluation of rectoanal coordination during simulated defecation [4]. High-resolution-anorectal manometry (HR-ARM) allows greater detail and resolution of the anorectal pressure change compared with conventional techniques [5]. Anatomical abnormalities and/or uncoordinated function of the rectum and anus can lead to faecal incontinence and/or symptoms of an evacuation disorder, with implications on quality of life.

There are few studies evaluating anorectal function in patients with inflammatory bowel disease (IBD). Most studies have used conventional manometry, and conclusions are limited by the inclusion of a heterogeneous cohort [6,7] of symptomatic patients with diverse indications for anorectal evaluation, active disease vs. inactive disease [7], and patients with CD ulcerative colitis [6,7,8,9]. Many studies have not included patients with perianal CD [8,10] or have only studied a small number of patients [11,12]. Other studies [6,7,9], with larger heterogeneous samples of IBD patients with active perianal disease, have not reported anorectal function according to perianal CD. There is a lack of information on the complications of healed/inactive perianal CD on anorectal function despite the prevalence and associated morbidity. Patients may be at risk of poor anorectal function due to excessive tissue damage. Crohn’s disease has also a high risk of recurrence [13].

Surgical treatment of perianal CD has the potential to compromise anal sphincter structure, which may predispose to subsequent faecal incontinence. The presence of symptoms associated with anorectal dysfunction, such as faecal incontinence, can sometimes poorly correlate with the presence of anal sphincter abnormalities, as shown in patients with obstetric anal sphincter injuries [14]. Moreover, even in patients without symptoms, the presence of anal sphincter abnormalities may have important implications for the future selection of the type of delivery [15] and might even pose a contra-indication for certain types of anorectal surgeries [16].

There is a need for a better understanding of the chronic complications of this disease and the possible role of HR-ARM and endoanal ultrasound (EAUS) in evaluating these. This pilot study aimed to evaluate anorectal function in treated and healed/inactive perianal CD.

## 2. Material and Methods

### 2.1. Study Design

This was a prospective cohort study conducted only in the Gastroenterology Department of St. James University Hospital, Leeds, UK, between April and December 2019. Patients attending the IBD Service were recruited. Patients were identified by AA in the outpatient clinic or by reviewing the medical records and offered participation if they met inclusion criteria. All patients provided written informed consent. Enrolled patients were given £50 to cover expenses. The study was approval by the North-West Greater Manchester East Research Ethics Committee, reference 18/NW/0850 and IRAS project number 255531. The study is registered at ClinicalTrials.gov (NCT03819257).

### 2.2. Inclusion and Exclusion Criteria

Patients aged 18 to 75 years in clinical remission defined by a Harvey-Bradshaw index <5 [17] were eligible if they had a perianal CD history defined by the presence of a perianal fistula (simple or complex perianal fistula) and/or abscess that was treated/healed/inactive. Current remission was confirmed by Perianal Disease Activity Index (PDAI) of ≤4 [18] and an EAUS (study procedure) without an image compatible with new/non-treated abscess or perianal fistula. Patients could be included regardless of the presence of absence of current anorectal symptoms. Current ileostomy or colostomy were exclusion criteria, as were vaginal delivery, previous haemorrhoidectomy, or lateral sphincterotomy, with these three as possible causes of sphincter injury. Previous or current anal fissure can limit EAUS performance (due to pain), have higher resting pressures on manometry, and could have been submitted to a previous surgery (e.g., lateral sphincterotomy) and therefore was also an exclusion criteria, as were an anal stricture and a current or previous rectovaginal fistula. Active rectal disease was also an exclusion criterion (histology). Absence of rectal involvement was defined by no previous rectal involvement ever described or, in the event of previous involvement, endoscopy within 18 months showing no current involvement. A previous seton placement, abscess drainage, or fistulotomy/lay open fistula were not exclusion criteria. Patients with current seton were only included if the seton was placed more than 24 weeks ago and if the EAUS did not show any new/non-treated perianal fistula/abscess.

### 2.3. Procedures

#### 2.3.1. Clinical Evaluation

An initial assessment (by interview) of symptoms suggesting obstructed defecation was conducted, namely prolonged and unsuccessful straining at stool, self-digitation for defecation, sense of incomplete evacuation or sensation of anorectal obstruction/blockage, and a stool frequency of less than three times per week. A clinical examination, including perianal inspection, palpation, and a digital rectal examination, was then performed to document any current fistulas with or without seton placement, possible anal strictures, and perianal scarring. A diagnosis of a functional defecation disorder could only be made in cases of compatible symptoms, negative balloon expulsion test, and an abnormal anorectal evacuation pattern on manometry [19].

#### 2.3.2. Water-Perfused High-Resolution Anorectal Manometry

All procedures were performed using a single-use anorectal catheter with 10 channels and an external diameter of 14Fr (Mui Scientific, Mississauga, ON, Canada) and by using the manometric system Solar GI HRM v9.5, Medical Measurement Systems (MMS), Enschede, the Netherlands. The balloons were non-latex and integrated with the manometric catheter. Patients lay in left lateral position with knees and hips bent at a 90° angle. No bowel preparation was given. The procedure was initially explained, and then, a lubricated probe was inserted into the rectum and remained stationary during the study. The most distal recording sensor was kept external to anal verge. We used the standardized protocol for HR-ARM as proposed by International Anorectal Physiology Working Group and the International Working Group for Disorders of Gastrointestinal Motility and Function [20] after a stabilization of 3 min:

1. One rest manoeuvre (60 s);
2. Three short squeezes (5 s) and an endurance squeeze (30 s)
3. Two single coughs
4. Three pushes (15 s)
5. Rectoanal inhibitory reflex (RAIR)
6. Rectal sensation for first sensation, desire to defecate, and maximum tolerate volume.

The reference values that were used for anal resting pressure, maximum anal squeeze increment, and cough pressure (greater of the three measurements) were previously validated by Rasijeff et al. [21] using a water-perfused HR-ARM in a UK population. For rectal sensation, the reference values used in the GI Physiology Unit in St. James Hospital are for first sensation, 10−40 mL; for desire to defecate, 70−120 mL; and for maximum tolerated volume, 140−250 mL. Values above these thresholds were considered hyposensitivity and below hypersensitivity. This evaluation was stopped at 250 mL. The disorders of the anorectal function were classified according to the recent London classification [20].

#### 2.3.3. Balloon Expulsion Test

With the patient resting in the left side position with hips and knees flexed, a non-latex balloon was inserted in the rectum after applying lubricating gel. This balloon was then filled with 50mL of tepid water. The patient was asked to sit on a commode and to try to expel the device in privacy, while the time was being recorded. The test ended when the patient expelled the balloon or when 3 min were reached. If the patient was unable to expel the device, the balloon was deflated and removed.

#### 2.3.4. 3D-Endoanal Ultrasound

All procedures were performed with a Hitachi ultrasound system using a 360° endoanal ultrasound probe EUP-R54AW-19 (Hitachi Medical Corporation, Tokyo, Japan). The patients were in the left lateral position, and no bowel preparation was given. The ultrasound probe was inserted in the anus to the upper anal canal. The visualization of the upper, middle, and lower anal canal was done for assessment of the internal and external anal sphincter integrity and fistulas/abscesses documentation. 3D-imaging capturing was performed in all patients. A fistula was considered healed if no image of a fistula was seen in the EAUS. A treated fistula was those with well-placed, current, or previous seton and a PDAI ≤ 4.

#### 2.3.5. Questionnaires

Harvey–Bradshaw index [17]: evaluates five items, namely general wellbeing, abdominal pain, number of liquid stools, presence of abdominal mass, and additional manifestations of CD. An index of <5 was considered suggestive of clinical remission.

Short IBD Questionnaire [22]: this questionnaire includes 10 questions evaluating the quality of life of patients with IBD.

Perianal Disease Activity Index—PDAI [18]: evaluates five items, including anal discharge, anal pain, restriction of sexual activity, type of perianal disease, and degree of induration. Each category is graded on a 5-point Likert scale ranging from no symptoms (score of 0) to severe symptoms (score of 4). A perianal PDAI of ≤4 was considered inactive disease.

Wexner score [23]: this is a five-item score, including solid, liquid, and gas incontinence; wearing pads; and lifestyle alterations. The items are scored from 0 (normal) to 4 (more severe).

### 2.4. Statistical Analysis

Continuous variables were described as mean (standard deviation). Categorical variables were described as frequencies. Statistical analysis was performed using SPSS software version 26 (SPSS Inc., Chicago, IL, USA).

## 3. Results

### 3.1. Study Cohort and Current Clinical Status

Sixteen patients were studied (mean age ± SD; 42 ± 13 years), of which 12 (75%) were male. According to the Montreal classification, most of the patients were classified as A2 (*n* = 9, 56%), L1 (*n* = 6, 38%), and B1 (*n* = 7, 44%). Three of the four women did not have any children, and one had a caesarean section. Only one (6%) patient had a history of previous proctitis (currently inactive). In total, 15 (94%) patients had a previous seton history, and four (25%) patients still had a seton(s) placed (Table 1). Twelve (75%) had at least one symptom suggestive of obstructed defecation, nine (56%) with a sense of incomplete evacuation, eight (50%) with straining, two (12%) needing digitation, and two (12%) with a sense of blockage. The mean Harvey–Bradshaw was 1.75 ± 1.52, with a minimum of 0 and maximum of 4 points. The PDAI median was 0 (IQR 0-2), with a minimum of 0 and maximum of 4 points. The mean short IBD questionnaire score was 57 ± 9 points, with a minimum of 34 and maximum of 68 points. There were four patients (25%) patients with faecal incontinence for solid and/or liquid stools. This information was provided in Table 1.

### 3.2. Endoanal Ultrasound

The EAUS showed a laceration and/or scarring of the internal anal sphincter in nine (56%) patients; information is provided in Table 2. Three cases were women, and six were men. There was no laceration in the external anal sphincter diagnosed. Four (25%) of patients had a fistula (s) with seton placement at least one year ago. One patient with a fistula had previously been treated with a seton, but this had now been removed. Five of nine (56%) patients with internal anal sphincter laceration and/ sphincter scarring had never experienced faecal incontinence for solid or liquid stools. All of the patients with faecal incontinence for solid or liquid stools had a laceration and/or scarring detected in the EAUS. The woman with a previous caesarean section had a normal EAUS. A woman considering having children had scarring and laceration of the internal anal sphincter.

**Figure 1:**
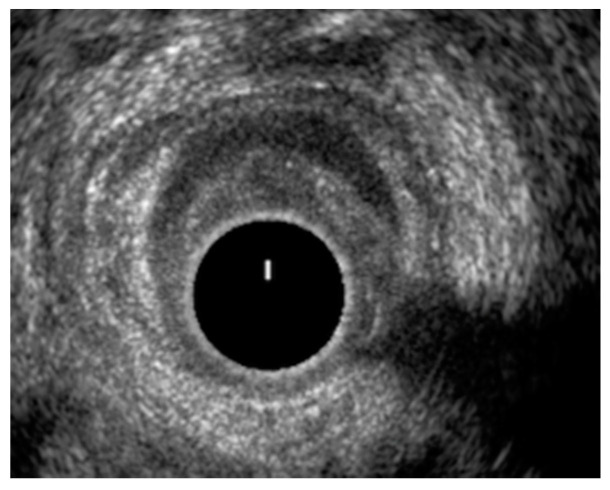
Laceration of the internal anal sphincter from 3 to 9 o’clock (left, posterior, right quadrant) in the middle anal canal. Transphincteric fistula with a seton in place, at 4–5 o′clock (left quadrant).

**Figure 2:**
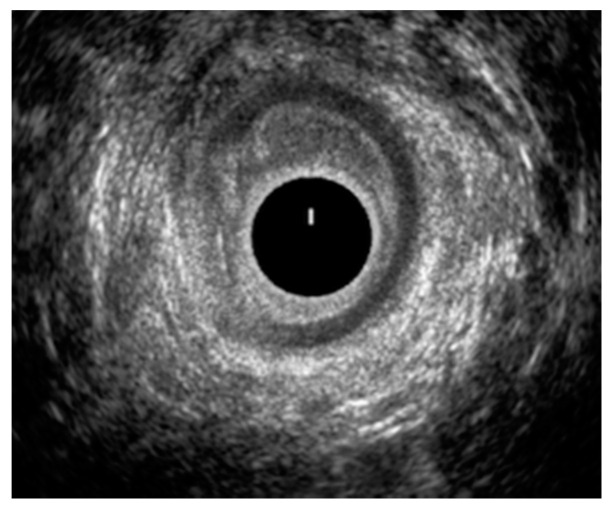
Laceration of the internal anal sphincter in the middle anal canal, between 7–11o’clock.

**Figure 3:**
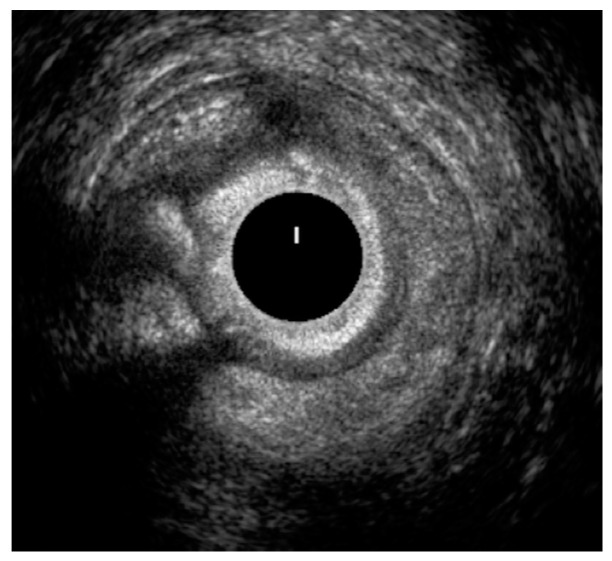
Integrity of the internal and external anal sphincters. Three fistulas identified, all with setons in place: transphincteric fistula at the anterior quadrant at 12 o’clock, right quadrant at 9 o’clock and posterior quadrant at 7 o’clock.

Figure 1 and Figure 2 show two EAUS of patients with internal anal sphincter laceration, with (Figure 1) and without (Figure 2) fistulas. Figure 3 is an EAUS from a patient with anal sphincter integrity and anal fistulas.

### 3.3. High-Resolution Anorectal Manometry and Balloon Expulsion Test

All patients had a normal average resting anal pressure (men: 80.0 ± 21.0 mmHg; women 71.1 ± 17.7 mmHg) and normal maximum anal squeeze pressure increment (men: 165.7 ± 89.6 mmHg; women: 80.9 ± 74.7 mmHg). The RAIR was present in all patients, and all had normal cough pressures, as shown in Table 2.

In five (31%) cases, the balloon expulsion test was negative; three of them had a manometry suggesting dyssynergia, and the other two had a normal rectoanal coordination. Only one (6%) patient had symptoms suggesting obstructed defecation, a negative balloon expulsion test, and manometric pattern of dyssynergia at the same time.

A manometric pattern suggesting dyssynergia was diagnosed in 11 (69%) patients. According to the London classification, eight (50%) cases had a normal expulsion with abnormal manometry, three (19%) cases with normal expulsion and normal manometry, two (12%) cases with abnormal expulsion and dyssynergia, two (12%) cases with abnormal expulsion and normal manometry, and one (6%) case of abnormal expulsion and poor propulsion dyssynergia.

Eleven (69%) of the patients had at least one out of the three sensory parameters (first sensation, desire to defecate, and maximum tolerate volume) suggesting hyposensitivity, and in 10/11 patients, the first sensation was above the normal threshold. All four patients with ongoing setons had hyposensitivity diagnosed in at least one of the sensory parameters. There were seven (44%) patients that had two of the sensory parameters, revealing hyposensitivity. Hypersensitivity in at least one parameter was seen in five (31%) cases and in all cases with an abnormal maximum tolerated volume. Only one (6%) patient did not have any disorder of rectal sensation at distension.

**Figure 4:**
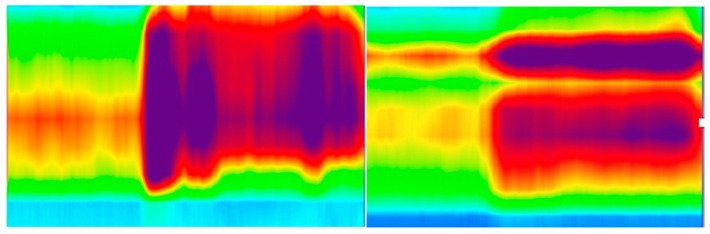
High-resolution manometry of two patients suggesting dyssynergia, both being unable to decrease anal pressure (relax) during the push manoeuvre.

Figure 4 are from two HR-ARMs suggesting dyssynergia.

## 4. Discussion

This was the first study systematically evaluating anorectal function on a homogeneous cohort of patients with treated/inactive perianal CD. There is a clear lack of information about the possible anorectal function complications from perianal CD, and studies including patients with healed/inactive perianal CD and stratifying the results accordingly were lacking. Perianal CD is especially prevalent in colonic disease and associated with a significant impairment of quality of life.

Endoanal ultrasound revealed scarring and/or internal sphincter laceration in more than half of patients. There were 56% of patients with ultrasonographical-defined anal sphincter injury. This clearly highlights the deleterious effect of surgical treatment of perianal CD. The absence of lacerations in the external anal sphincter was probably related to the exclusion of women with a history of previous vaginal delivery. Interestingly, many of the patients with sphincter abnormalities had never experienced episodes of faecal incontinence for solid or liquid stools. Our most important finding is that the absence of faecal incontinence does not preclude the absence of sphincter injuries. In women with inactive perianal CD, knowing the anal sphincter’s integrity before the mode of delivery might be relevant regardless of faecal incontinence. In our cohort, there was one woman with a previous history of an anal fistula in the six years before, and her EAUS revealed scarring and a laceration in the internal anal sphincter. The performance of a planned caesarean has been suggested [24] in IBD cases of (1) women with high-risk of obstetric injuries, (2) active perianal disease, or (3) ileal pouch-anal anastomosis. There are no guidelines in how to proceed in cases of previous/inactive perianal CD. The role of EAUS in women with a previous history of perianal CD (inactive) to evaluate anal sphincters and to help guide the mode of delivery should be further explored.

A study by Anderson et al. [10] comparing the anorectal function of CD without endoscopic proctitis and without perianal disease and healthy controls showed rectal hypersensitivity in CD patients. Results are somewhat conflicting with a study by Muller et al. [11], also comparing CD without macroscopic rectal inflammation and healthy controls, showing a higher volume threshold for first sensation in CD (statistically significant between groups), with a first sensation for controls at a mean volume of 37.5 mL vs. 57.9 mL for CD. Studies with CD patients with macroscopic and/or microscopic proctitis have reported rectal hypersensitivity [12]. This type of alterations can be associate with faecal incontinence [12]. The London Classification [20] states hyposensitivity is defined as “≥2 out of 3 sensory parameters >ULN”, whereas hypersensitivity is defined as “1 or more sensory parameter(s) <LLN” as a major finding. If these definitions were applied to the current data set, seven (44%) subjects would be diagnosed with hyposensitivity and five (31%) with hypersensitivity. As a pilot study with a small sample, we could not provide a pathophysiologic explanation for this, but it might be that the fibrosis induced by the healing and seton placement could be associated with rectal hyposensitivity. These findings need to be explored in further studies.

In total, 11 patients had abnormal anorectal coordination at manometry. A previous study by Litta et al. [7], including both CD and ulcerative colitis with perianal complaints, showed a similar prevalence of a manometric dyssynergic pattern, but that did not differ from healthy controls. There are other studies showing a high percentage of a dyssynergic pattern in healthy controls [25]. This might be related to the manometric limitations for this evaluation, namely the fact that the patient is not in the normal position for defecation, a poor understanding of the manoeuvre, and some patients might not fully comply due to fear of having an accident [7]. The diagnosis of a defecation disorder requires the combination of suggestive symptoms and two positive tests, e.g., an anorectal manometry and a negative balloon expulsion test [19]. In our cohort, there was a high number of patients with symptoms and a dyssynergic manometric pattern, but only one patient met criteria for the diagnosis of a defecation disorder.

Our study has several strengths, namely the strict exclusion criteria. We have excluded women with previous vaginal delivery, which can be itself a major cause of sphincter laceration and anorectal dysfunction, and also patients with previous haemorrhoidectomy or lateral sphincterotomy. Patients with active proctitis were also excluded. A previous study by Chrysos et al. [12] showed that microscopic (histological) rectal involvement in CD patients, even in the absence of endoscopic involvement, was associated with anorectal disfunction, namely lower resting and squeeze pressure and rectal hypersensitivity. We used the very recently published London classification for the disorders of the anorectal function, which has standardized the performance and interpretation of anorectal function tests [20]. Reference values for anal pressures were previously validated in a UK population using water-perfused HR-ARM [21].

There were several limitations. This was a pilot study with small sample size and no control group. However, for a small sample, the results are very consistent. To avoid detecting obstetric injuries that could be mistaken for the results of perianal CD, we excluded women with a vaginal delivery, and as a result, we had a male-dominated study cohort, and the small group of females may not be representative of the wider female IBD population. The lack of a control group can be somewhat mitigated by the use of reference values reported to water-perfused HR-ARM in a UK population [21]. Sphincter lacerations or scarring are not to be expected in healthy young individuals without previous vaginal delivers or anorectal surgeries. A more in-depth analysis of the relationship between the duration of the seton position, type (classification) of fistulas, and anorectal abnormalities was impaired given the lack of precise information on the patients records, especially in cases when the seton(s) might have been in place for several years, placed many years ago, or became dislodged spontaneously. There were five patients with current anal fistulas, but all have been treated with biologics and seton placement, with clear improvement and a PDAI ≤4 points, which was considered inactive disease [18]. We did not use other additional methods for evaluate rectal sensation, e.g., barostat, as this is not part of routine clinical practice for evaluation.

## 5. Conclusions

This was the first study evaluating anorectal function in a homogeneous cohort of patients with treated perianal CD by using anorectal HR-ARM and EAUS. More than half of the patients had an EAUS showing scarring and/or laceration in the internal anal sphincters, but many remained asymptomatic, and all cases showed normal resting pressure. The role that EAUS plays in the evaluation of sphincter integrity in specific groups of asymptomatic patients, e.g., in women with inactive perianal CD, to help guide the selection of the mode of delivery needs to be further studied. Symptoms of and an abnormal anorectal coordination were frequently seen in the HR-ARM, but a diagnosis of defecation disorder was uncommon. Rectal hyposensitivity as a possible complication of the fibrosis induced by the healing of perianal CD, and the impact that setons might have in this rectal (hypo) sensitivity also needs to be further explored. This pilot study brings new information in this type of cohort and, more importantly, unveils a direction for future larger studies.

## Figures and Tables

**Table 1:** Patient description.

Patient	Gender	Montreal Classification	Previous Proctitis	Previous Seton	Ongoing Seton	Obstructed Defecation Symptoms (Any)	Harvey– Bradshaw (Points)	PDAI (Points)	Quality of Life (Points)	Faecal Incontinence for Solid or Liquids	Wexner Score (Points)
1	Male	A2 L3 B2p	No	Yes	Yes	Yes	3	3 ^#^	66	No	0
2	Male	A1 L1 B2p	No	Yes	Yes	No	4	3 ^#^	34	No	0
3	Female	A3 L1 B2p	No	No	No	No	4	1	62	Yes	4
4	Male	A1 L3 + 4 B1p	No	Yes	No	Yes	0	0	64	No	0
5	Male	A2 L2 B1p	No	Yes	No	Yes	0	0	57	Yes	1
6	Male	A2 L1 B2p	No	Yes	Yes	Yes	2	4 *	53	No	1
7	Male	A1 L3 B2p	Yes	Yes	No	Yes	1	0	51	No	0
8	Female	A2 L2 B2p	No	Yes	No	Yes	1	0	49	No	0
9	Male	A2 L3 B3p	No	Yes	No	Yes	2	0	66	No	0
10	Male	A1 L3 B1p	No	Yes	No	Yes	0	0	68	No	1
11	Male	A3 L2 B1p	No	Yes	Yes	No	3	2	60	No	0
12	Male	A2 L1 B3p	No	Yes	No	No	4	0	57	Yes	1
13	Male	A2L1B1	No	Yes	No	Yes	0	0	54	No	0
14	Male	A3L2B1	No	Yes	No	Yes	2	2	42	No	0
15	Female	A2L2B1	No	Yes	No	Yes	0	0	61	Yes	6
16	Female	A2L1B3	No	Yes	No	Yes	2	0	65	No	0

*: 3 of the 4 points were due to 3 fistulas that were seen. ^#^: 2 of the 3 points were due to 2 fistulas that were seen. PDAI, perianal disease activity index.

**Table 2:** Balloon expulsion test, anorectal manometry, and endoanal ultrasound results.

Patient	Balloon Expulsion Test	Scaring or Laceration EAUS		Anorectal Manometry							
			Average Anal Resting Pressure	Maximum Anal Squeeze Pressure Increment	Cough Pressure	Sensation			Rectoanal Coordination	Rectoanal Inhibitory Reflex	London Classification for Rectoanal Coordination
						First Sensation	Desire to Defecate	Maximum Tolerate Volume			
1	No	No	Normal	Normal	Normal	Hypo	Hypo	Hypo	Abnormal	Present	Abnormal expulsion Dyssynergia
2	No	Yes	Normal	Normal	Normal	Hypo	Hypo	Hypo	Abnormal	Present	Abnormal expulsion Dyssynergia
3	Yes	Yes	Normal	Normal	Normal	Hypo	Hypo	Normal	Abnormal	Present	Normal expulsion Abnormal manometry
4	Yes	No	Normal	Normal	Normal	Hypo	Normal	Normal	Abnormal	Present	Normal expulsion Abnormal manometry
5	Yes	Yes	Normal	Normal	Normal	Hypo	Hypo	Normal	Abnormal	Present	Normal expulsion Abnormal manometry
6	Yes	No	Normal	Normal	Normal	Normal	Hypo	Hypo	Normal	Present	Normal expulsion Normal manometry
7	Yes	Yes	Normal	Normal	Normal	Normal	Hyper	Hyper	Abnormal	Present	Normal expulsion Abnormal manometry
8	Yes	No	Normal	Normal	Normal	Hypo	Normal	Normal	Abnormal	Present	Normal expulsion Abnormal manometry
9	Yes	Yes	Normal	Normal	Normal	Hypo	Hypo	Normal	Abnormal	Present	Normal expulsion Abnormal manometry
10	No	Yes	Normal	Normal	Normal	Hypo	Hyper	Hyper	Normal	Present	Abnormal expulsion Normal manometry
11	Yes	No	Normal	Normal	Normal	Hypo	Normal	Normal	Abnormal	Present	Normal expulsion Abnormal manometry
12	No	Yes	Normal	Normal	Normal	Normal	Hyper	Hyper	Abnormal	Present	Abnormal expulsion Poor propulsion and dyssynergia
13	Yes	No	Normal	Normal	Normal	Normal	Normal	Hyper	Normal	Present	Normal expulsion Normal manometry
14	Yes	No	Normal	Normal	Normal	Hypo	Hypo	Normal	Abnormal	Present	Normal expulsion Abnormal manometry
15	No	Yes	Normal	Normal	Normal	Normal	Normal	Hyper	Normal	Present	Abnormal expulsion Normal manometry
16	Yes	Yes	Normal	Normal	Normal	Normal	Normal	Normal	Normal	Present	Normal expulsion Normal manometry

EAUS, endoanal ultrasound.

## Data Availability

The datasets used and/or analysed during the current study are available from the corresponding author on reasonable request.
